# The Relationship between the Phases of the Menstrual Cycle on the Incidence and Severity of Headache after Spinal Anesthesia

**DOI:** 10.1055/s-0039-1696967

**Published:** 2019-09-24

**Authors:** Mahmoud reza Moradkhani, Arash Karimi, Zahra Zarei, Sepideh Vahabi

**Affiliations:** 1 Department of Anesthesiology, Faculty of Medicine, Lorestan University of Medical Sciences, Khorramabad, Iran; 2 Student of Research Committee, Lorestan University of Medical Sciences, Khorramabad, Iran

**Keywords:** postdural puncture headache, spinal anesthesia, menstrual cycle

## Abstract

**Introduction** Headache after spinal anesthesia is a common complication, which is caused after dural puncture due to discharge of cerebrospinal fluid and reduction in the volume and pressure. Studies have shown that a variety of factors are involved including needle shape, needle size, patient's sex, age, duration of surgery, history of spinal anesthesia, and history of headaches. One possible factor is the phase of the menstrual cycle. Many studies have investigated the effect of the menstrual cycle on factors such as postoperative nausea, vomiting, propofol injection pain, and sore throat after intubation. Also, many studies have investigated the effect of different phases of the menstrual cycle on migraine headaches. Therefore, we decided to investigate the effect of different phases of the menstrual cycle on headache after spinal anesthesia.

**Materials and Methods** To determine the relationship between headache after spinal anesthesia and menstrual cycle, the study included all the patients undergoing spinal anesthesia in Shohada Ashayer and Asalian Hospitals of Khorramabad. This cohort study included 279 patients, and data collection tool was a questionnaire. The data from the questionnaire included age, menstrual phase, surgical procedures, pain location, pain intensity, history of spinal anesthesia, history of headache, and headache after spinal anesthesia.

**Results** There was no statistically significant difference between the location of headache, the history of spinal anesthesia, the location of headache, the history of headache, and menstrual phase.

**Conclusion** Considering the high incidence of postdural puncture headache in follicular phase, it is recommended that patients with a high risk of headaches undergo spinal anesthesia and surgery in the luteal phase.

Spinal anesthesia is one of the common anesthetic methods that is a subset of regional anesthesia. This method is easy to perform, has a rapid effect, and has good pain control during surgery. 1 Its complications include nausea, back pain, headaches, tinnitus, acute hearing loss, hypotension, bradycardia, apnea, hypoxia, hypo ventilation, urinary retention, and vomiting. 1 2

Headache is a common complication after spinal anesthesia, due to an outflow of cerebrospinal fluid and reducing the volume and pressure, followed by meningeal vessels and nerves, in other painful parts, and on the other hand to maintain intracranial volume, intracranial blood vessels dilation, which causes headaches. 2 This headache has a pulsating nature; it is exacerbated by sitting and is better when sleeping; it is felt more in the forehead and behind the head and in severe cases with symptoms such as neck stiffness, shoulder pain, low back pain, hearing loss, tinnitus, dullness, dizziness nausea, and vomiting. 3 The headache falls in 24% of cases within 48 hours, more than 50% in 4 days and 72% in 1 week. Studies have showed that various factors such as needle shape, size, sex and age of the patient, duration of surgery, spinal anesthesia history, and headache history play an important role in complications. 2 4 One of the probable factors of the various phases of the menstrual cycle.

The menstrual cycle is the natural reproductive age of women, with monthly rhythmic changes in the level of secretion of female hormones and the corresponding changes in the ovaries and sexual organs, called the female sexual cycle. This cycle is on average 28 days and the ovulation is on the 14th day. The first day of bleeding is considered as the first day of the new menstrual cycle. At the endometrial level, the menstrual cycle consists of four main stages: (1) menstrual bleeding, (2) proliferative stage, (3) ovulation stage, and (4) secretion

At the ovary level, the menstrual cycle has two main stages: (1) follicular stage (first half) and (2) luteal stage (second half).

Many studies have shown that the menstrual cycle has a significant effect on factors such as postoperative nausea and vomiting, pain injecting propofol, and throat irritation after intubation is done.

Many studies have also been done on the effective role of various phases of the menstrual cycle in migraine headaches. 5 6 Considering the above studies, we decided to examine the effect of different phases of menstrual cycle on headache after spinal anesthesia.

## Materials and Methods

This is a cohort study were 279 women who have regular cycles were entered into the study at Shohada Ashayer and Asalian Hospitals of Khorramabad. This study was conducted during the summer and autumn of 2014.

*Sampling* : Eligible patients were enrolled to the desired number of samples at the beginning of the study under spinal anesthesia.

Inclusion criteria included women aged between 16 and 45 years, having regular menstrual, nonuse of contraceptives, nonpregnant patients, completion of a written consent by the patient participating in the study, American Society of Anesthesiologists (ASA) class 1 and 2, lack of spinal infection, lack of coagulation disorders, and lack of breastfeeding.

Data were collected by questionnaire, the patients were followed for a week, occurrence and severity of headache they observed were asked and recorded. History of the patient's menstrual cycle and previous menstrual cycle is considered the first day of operation on account of the time, and patients were divided into two groups: The beginning of the follicular phase of the cycle until the start of the luteal phase and the luteal phase day 14 until the last day.

### Method of Anesthesia

After transferring the patient to the operating room, venipuncture was performed and intravenous Ringer's lactate solution (3–5 cc per kg of body weight within 15 minutes) was infused to the patient. Then, spinal needle (No. 25, from Spinocan, Indonesia) was inserted in a sitting position, and 0.5% Marcaine (the cc4) from Bupivacaine MYLAN was administered, this was performed by one anesthesiologist, and if the spinal anesthesia was performed on a particular patient more than once, the patient was excluded from the study. Ephedrine 5 mg intravenously and atropine 5 μg/kg were given to the patients after spinal anesthesia. After operation, the incidence and severity of headaches and spinal action were evaluated.

*For headaches* : incidence of severity was observed during surgery, in-time recovery, in hours: 6, 12, 18, and 24 first and second day, and in the third and seventh days if registering by telephone two times a day (at 10 am and 10 pm) were followed. Pain by visual analog scale on the basis of scores from 0 to 10 was used. A score of 0 means there is no pain, and 10 shows the worst possible pain score. Patients with pain score greater than 5 were treated with acetaminophen codeine pro re nata, and if there is no response to treatment or pain score greater than 7, treatment with 1 mg/kg meperidine was done.

### Statistical Analysis

The results were analyzed in terms of relative risk and confidence interval associated with contingency tables, and chi-squared test. Pain severity was compared using the Mann–Whitney U test. A *p* -value <0.05 was considered significant.

## Results

All information obtained from the questionnaires, such as the type of surgery, the phase of the menstrual cycle, headaches, and migraine history, was assessed, the results of which are as follows.

Of the 279 patients studied, most of the headaches were related to patients in the follicular phase ( *p* < 0.010) ( Table 1 ).

**Table 1:** Distribution of subjects based on the menstrual phase and the risk of postdural puncture headache

Headache/menstrual phase	Headache		No headache		Total
	Number	Percentage	Number	Percentage	
Follicular phase	21	8.15%	112	91.85%	133
Luteal phase	9	2.6%	137	97.4%	146
Total	30	10.8%	249	89.2%	279
*p* < 0.010 and χ ^2^ = 6.7.					

Headache intensity during surgery was 0.55 and recovery time was 0.0 ( *p* < 0.09). Headache intensity on the first day in the follicular and luteal phase was 0.28 and 0.23, respectively ( *p* < 0.00), and on the second day, it was 1.02 and 0.45 ( *p* < 0.66), respectively ( Table 2 ).

**Table 2:** Evaluation of moderate-intensity headache after spinal anesthesia in subjects in the first and second day

Headache duration/second day menstrual phase	6 h	12 h	18 h	24 h	Total
Follicular phase	0.14	0.14	0.38	0.46	0.28
Luteal phase	0.05	0.22	0.22	0.44	0.23
*p* < 0.000 and χ ^2^ = 14.6					
Follicular phase	0.4	0.89	0.9	1.9	1.02
Luteal phase	0.3	0.26	0.54	0.7	0.45

There was a significant difference in the duration of headache between the follicular and luteal phase on the third to seventh day ( *p* < 0.021; 0.015; 0.011; 0.55; and 0.07), respectively ( Table 3 ). Severe headache in the follicular and luteal phase was 2.81 and 1.23 in the first week after spinal anesthesia, and the highest average score in the third, fourth, and fifth severe headache was significantly greater in the follicular than luteal phase.

**Table 3:** The average severity of headache after spinal anesthesia in the patients studied in the third to seventh day

Third day/Menstrual phase	10 am	10 pm	Total
Follicular phase on day 3	6.43	5.92	6.13
Luteal phase on day 3	2.03	2.67	2.35
*p* < 0.021 and χ ^2^ = 6.4			
Follicular phase on day 4	5.33	6.01	5.67
Luteal phase on day 4	3.01	3.11	3.06
*p* < 0.015 and χ ^2^ = 3.99			
Follicular phase on day 5	5.1	6.9	6
Luteal phase on day 5	2.9	2.6	2.75
*p* < 0.011 and χ ^2^ = 4.59			
Follicular phase on day 6	2.9	1.7	2.3
Luteal phase on day 6	0.9	0.8	0.85
*p* < 0.055 and χ ^2^ = 5.09			
Follicular phase on day 7	0.7	0.5	0.6
Luteal phase on day 7	0.3	0	0.15
*p* < 0.07 and χ ^2^ = 7.4			

Type of surgery in the follicular phase is as follows: 59% hemorrhoids, 31% pilonidal sinus, 7% appendectomy, and 3% Fisher's exact test, while in the luteal phase, type of surgery is 87% hemorrhoids, 11% pilonidal sinus, and 2% appendectomy. There was no statistically significant difference in the type of surgery performed ( *p* < 0.98).

Position of headache in the follicular was significantly different from those in the luteal phase ( *p* < 0.00) ( Table 4 ). The duration of headache in the follicular phase: 4.5% in first day to third day, and 11.3% during the fourth to seventh day, while in the luteal phase, the duration of headache: 1.4% in first days to third day and 4.8% in the fourth to seventh day ( *p* < 0.00) ( Table 5 ).

**Table 4:** Frequency distribution of subjects according to the menstrual phase and the incidence of PDPH

Headache/Menstrual phase	Forehead		Behind		The parietal		Total head		Total
	Number	%	Number	%	Number	%	Number	%	
Follicular phase	21	15.8	0	0	0	0	0	0	21
Luteal phase	2	1.4	7	4.8	0	0	0	0	9
Total	23	8.2	7	2.5	0	0	0	0	30
*p* < 0.00 and χ ^2^ = 24.6.									

Abbreviation: PDPH, postdural puncture headache.

**Table 5:** Frequency distribution of subjects according to the menstrual phase and duration of PDPH

Phase menstrual/headache duration	1 to 3 days		3 to 7 days		Total
	Number	%	Number	%	
Follicular phase	6	4.5	15	11.3	21
Luteal phase	2	1.4	7	4.8	9
Total	8	2.9	22	7.9	30
*p* < 0.00 and χ ^2^ = 6.82.					

Abbreviation: PDPH, postdural puncture headache.

In individuals with a history of migraine headache after spinal anesthesia with 38% in the follicular phase and 33% in the luteal phase were not significantly different ( *p* < 0.81).

Positions in the follicular phase during spinal anesthesia: 38% supine, 14% Peron, 47% semi-sitting, and in the luteal phase: 22% supine, 11% Peron, 66% semi-upright, which was not significantly difference ( *p* < 0.21).

## Discussion and Conclusion

In the present cohort study conducted at Shohada Ashayer and Asalian Hospitals of Khorramabad, 279 eligible patients were enrolled and demographic characteristics such as age distribution, phase of the menstrual cycle, type of surgery, pain, location of pain, and migraine headaches were evaluated.

In this study, the incidence of headache was higher in the follicular phase compared with the luteal.

About 8.15% patients in the follicular phase and 2.6% in the luteal phase had headaches, and the intensity of the headache in the follicular phase is higher than the luteal phase.

A study was conducted by Echevarria et al 7 about spinal headache on 100 women with regular menstrual cycle based on the time of the sexual cycle; they were divided into two groups: postmenstrual and premenstrual. Seven (3.4%) patients with premenstrual and six patients with postmenstrual cycle had postdural puncture headache (PDPH), with ineffective menstrual cycle and hormone levels with spinal headache. In the present study, 30 patients were PDPH, while 21 patients in the follicular phase and 9 in the luteal phase were identified with headache, which was significantly higher in the follicular phase ( *p* < 0.010).

According to a study conducted by Mohammad et al on the factors associated with headache after spinal anesthesia, 200 patients aged between 15 and 65 years, with ASA class 1 and 2 had headaches of 6. 5, 5.3, and 1% in day 1, a week and a month after spinal anesthesia, respectively. The rate was higher in patients with a history of spinal anesthesia. The incidence of headache was lower in our study as compared with Haqiqi et al's 12 study on both sexes, but our study was conducted on young women with a risk factor for the occurrence of PDPH. The high incidence of headache in the first week of our study is similar, but compared with other reviews, we have a much lower incidence of headache on the first day, which is not consistent with the results of Haqiqi et al ( *p* < 0.00). In a study by Jabbari et al, 8 conducted on 200 patients between 19 and 30 years during the three postoperative visits, there was a high incidence of spinal headache, 7 (3.5%) were suffered from PDPH, and 3 (1.5%), suffered from tension haddock. The former study shows a young woman and increases pregnancy rates, while, in our study of 279 patients, 30 patients had PDPH which was significantly higher than in the study conducted by Jabbari et al. In addition, all patients were female and of childbearing age. The incidence of headache is much higher than in the study conducted by Jabari et al.

A study conducted by Hanci et al 9 was about the effect of menstruation on painful propofol injection on 160 regular menstruating women in the second phase of the follicular and luteal phase sexual cycle. The menstrual cycle was clearly influenced by the amount of pain during propofol injection, and its rate was lower in the luteal phase. In our study, which evaluated PDPH, the results were similar to this study and showed that the incidence of headache in the follicular phase was higher and lower in the luteal phase; it seems that the hormonal changes created during the menstrual cycle in the pain experience are similar in all respects. According to study by Sener et al, 10 the effect of the menstrual cycle on postoperative nausea and vomiting on 111 women aged between 18 and 35 years, in 3 groups including the follicular, eyelid, and luteal phase. Results showed that nausea and vomiting were less in the luteal phase, while in our study we looked at the headache after spinal anesthesia but the results showed similarly with this study, the incidence of headache in the follicular phase was less in the luteal phase.

A study conducted by Hellström and Anderberg 11 was on the influence of cycle on the perception of pain after surgery on 80 women with a regular menstrual cycle; the study was based on the time of the sexual cycle and study groups were divided into two groups: luteal phase and follicular phase. A significant difference in pain perception in the different phases was not observed, whereas in our study the effect of PDPH on headache in the follicular phase was higher; perhaps this is due to the severity of postoperative pain at the site of surgery that is difficult for the patient to differentiate the pain of menstrual phases.

According to studies by Macgregor, 13 the effect of the menstrual cycle on migraine headache was performed on 81 patients with a clinical diagnosis of migraine. The results showed that migraine headache increased significantly in the first 2 days of menstruation and the least amount of headache was during ovulation. In our study, which evaluated PDPH, out of 30 patients, 8 patients had a history of follicular phase migraine and 3 patients the luteal phase migraine, and there was no significant difference in the severity of headache in different phases of menstruation. In this study, the incidence of headache in the follicular phase was higher than that of the luteal. About 15.8% of patients had a follicular phase headache and 6.2% of them had the luteal phase headache. Considering the high incidence of PDPH in follicular phase, it is recommended that patients with a high risk of headaches undergo spinal anesthesia and surgery in the luteal phase.
